# 18 years of results with cemented primary hip prostheses in the Norwegian Arthroplasty Register: Concerns about some newer implants

**DOI:** 10.3109/17453670903161124

**Published:** 2009-08-01

**Authors:** Birgitte Espehaug, Ove Furnes, Lars B Engesæter, Leif I Havelin

**Affiliations:** ^1^The Norwegian Arthroplasty Register, Department of Orthopaedic Surgery, Haukeland University HospitalBergenNorway; ^2^Department of Surgical Sciences, University of BergenBergenNorway

## Abstract

**Background and purpose** Few studies have compared the long-term survival of cemented primary total hip arthroplasties (THAs), and several prostheses have been used without adequate knowledge of their endurance. We studied long-term outcome based on data in the Norwegian Arthroplasty Register.

**Patients and methods** The 10 most used prosthesis brands in 62,305 primary Palacos or Simplex cemented THAs reported to the Register from 1987 through 2007 were included. Survival analyses with revision as endpoint (for any cause or for aseptic loosening) were performed using Kaplan-Meier and multiple Cox regression with time-dependent covariates. Revision rate ratios (RRs) were estimated for the follow-up intervals: 0–5, 6–10, and > 10 years.

**Results** 5 prosthesis brands (cup/stem combinations) (Charnley, Exeter, Titan, Spectron/ITH, Link IP/Lubinus SP; n = 24,728) were investigated with 0–20 year follow-up (inserted 1987–1997). After 18 years, 11% (95% CI: 10.6–12.1) were revised for any cause and 8.4% (7.7–9.1) for aseptic loosening. Beyond 10 years of follow-up, the Charnley cup had a lower revision rate due to aseptic loosening than Exeter (RR = 1.8) and Spectron (RR = 2.4) cups. For stems, beyond 10 years we did not find statistically significant differences comparing Charnley with Titan, ITH, and SP stems, but the Exeter stem had better results (RR = 0.5). 10 prosthesis brands (9 cups in combination with 6 stems; n = 37,577) were investigated with 0–10 years of follow-up (inserted from 1998 through 2007). The Charnley cup had a lower revision rate due to aseptic loosening than all cups except the IP. Beyond 5 years follow-up, the Reflection All-Poly cup had a 14 times higher revision rate. For stems, beyond 5 years the Spectron-EF (RR = 6.1) and Titan (RR = 5.5) stems had higher revision rates due to aseptic loosening than Charnley. The analyses also showed a marked improvement in Charnley results between the periods 1987–1997 and 1998–2007.

**Interpretation** We observed clinically important differences between cemented prosthesis brands and identified inferior results for previously largely undocumented prostheses, including the commonly used prosthesis combination Reflection All-Poly/Spectron-EF. The results were, however, satisfactory according to international standards.

## Introduction

A systematic review of the literature concerning outcome and clinical effectiveness of prostheses used for primary total hip arthroplasty (THA) showed that among the many cemented prostheses in use in Norway in 2000, only the Charnley and the Lubinus IP prosthesis had been reported with results beyond 15 years of follow-up (Aamodt et al. 2004). Several of the prostheses in common use today have insufficient published documentation of clinical quality, or lack it altogether. We compared the survival of the 10 most used prosthesis brands as reported to the Norwegian Arthroplasty Register during the years 1987 to 2007.

## Material and methods

### The Norwegian Arthroplasty Register (NAR)

The NAR was established September 15, 1987 (Havelin 1999, Havelin et al. 2000). Individual reports of THAs have since been received from 86 orthopedic departments performing this procedure. Information on primary operations and revisions, including the identity of the patient, the date of operation, indication, type of prosthesis and cement, is reported on a standardized form by the orthopedic surgeon. An English translation of the form can be found on the register's website at http://www.haukeland.no/nrl. About 98% of all total hip replacements are reported to the register (Espehaug et al. 2006). Implant failure is defined as the surgical removal or exchange of the whole or part of the implant. Linkage of information on the primary operation and subsequent revisions is possible by use of the unique identification number assigned to each resident of Norway.

### Study sample

**Figure 1.**
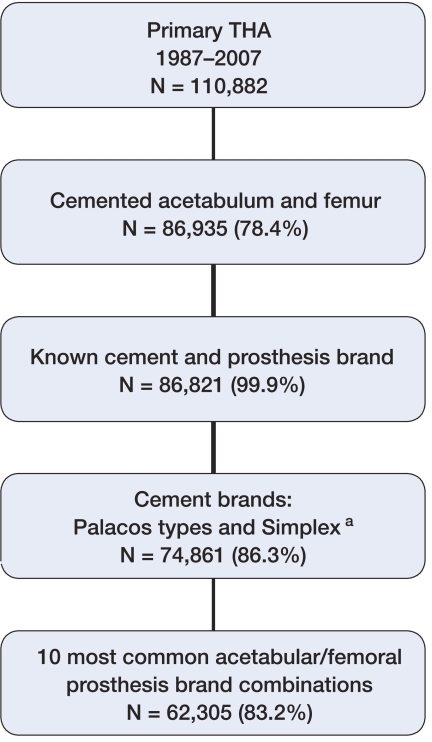
Description of the study selection procedure. **^a^** Palacos cement types: Palacos plain, Palacos with gentamycin, Refobacin – Palacos, Palacos R + G, and Refobacin Bone Cement R, and Simplex cement: Simplex plain, Simplex with erythromycin/ colistin, and Simplex with tobramycin. Same cement brand in acetabulum and femur.

By December 31, 2007, 110,882 primary THAs had been reported to the Register, 8,094 of which (7.3%) were revised by 2007. Only THAs with cemented acetabular and femoral components were eligible for inclusion in the present study (78%). Furthermore, THAs with unknown information on prosthesis brand or cement brand (n = 114), or on whether the components were cemented with two different cement brands (n = 2,373), were excluded. An exclusion criterion was also that the prosthesis components should be cemented with either a Palacos type cement (Palacos plain, Palacos with gentamycin, Refobacin-Palacos, Palacos R+G, or Refobacin Bone Cement R), or a Simplex cement (Simplex plain, Simplex with erythromycin and colistin, or Simplex with tobramycin) (n = 74,861). Furthermore, only the 10 most common prosthesis brand combinations were studied, totaling 62,305 THAs (Figure 1).

### Statistics

**Figure 2.**
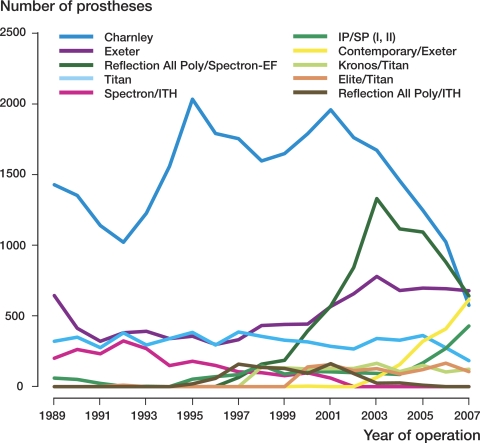
Time trends in the use of the 10 most common cemented prosthesis brands in Norway 1989–2007.

Survival analyses used revision of either cup or stem, revision of cup, or revision of stem as endpoints. Separate analyses were performed for revisions for any cause and revisions due to aseptic loosening. Information on deaths or emigrations was retrieved from Statistics Norway, Oslo, until December 31, 2007. The survival times of implants in patients who had died or emigrated without revision of the prosthesis were censored at the date of death or emigration. Survival times of unrevised prostheses were otherwise censored at the end of the study on December 31, 2007. Use of prosthesis brands changed throughout the study period (Figure 2). To ensure that prosthesis brands were compared within the same time period, separate analyses were performed for THAs done before 1998 (with follow-up through 2007) and from 1998 through 2007. For the first time period, we studied 5 prosthesis combinations that had been used in more than 250 hips (5 different cups and 5 different stems). For the second time period, all 10 prosthesis combinations were studied (9 different cups and 6 different stems).

Overall revision percentages were estimated using the Kaplan-Meier method. Median follow-up was calculated for each prosthesis brand following the reversed Kaplan-Meier method (Schemper and Smith 1996). Cox regression analyses with prosthesis brand as stratification factor were used to construct prosthesis-specific survival curves adjusted for sex, age (< 60, 60–69, 70–79, > 79), diagnosis (osteoarthritis, other), use of systemic antibiotic prophylaxis (no, yes) and cement brand (8 brands). In the Cox models, the covariate age was represented with indicator variables since the assumption of a log-linear relationship between age and the revision rate was not justified. The survival curves for the adjusted percentage of unrevised implants were constructed for times when more than 50 implants remained at risk of revision. Adjusted revision rate ratios (incidence rate ratios) (RRs) for the different prosthesis brands are presented with 95% confidence intervals and p-values relative to the Charnley prosthesis. Charnley was chosen as a reference because it was used in large numbers throughout the study period. To investigate the proportional hazards assumption of the Cox model (meaning that the relative difference between revision rates should be constant over time since the primary operation), we used tests and visual inspection of plotted scaled Schoenfeld residuals (Grambsch et al. 1995). These analyses showed that some of the prosthesis brands did not satisfy this assumption. Adjusted revision rate ratios were therefore established also within time intervals (0–5 years, 6–10 years, and > 10 years after the primary operation) using an extended Cox model including time-dependent covariates. The time-dependent covariates were based on heavy side functions with cut-points at 5 and 10 years.

To investigate whether the adjusted log RRs changed with year of operation, we fitted an extended Cox model based on generalized additive models for survival data with penalized splines (Hastie and Tibshirani 1990). This smoothing method makes no assumptions about the shape of the association, and therefore permits estimation of non-linearities. The graphs (Figure 6) were calibrated so that the log RR was set to zero at the mean year of operation. A horizontal line was added to show this reference level. The graphs are presented with 95% confidence intervals, together with the results of a linear trend test and a test of non-linearity in the effect of year of operation on survival. To ensure at least 5 years of follow-up, only THAs operated before 2003 were included in this analysis.

All p-values less than 0.05 were considered statistically significant. The statistical software programs S-Plus 7 (Insightful Corp., Seattle, WA) and SPSS version 15.0 (SPSS Inc., Chicago, IL) were used.

## Results

**Figure 3.**
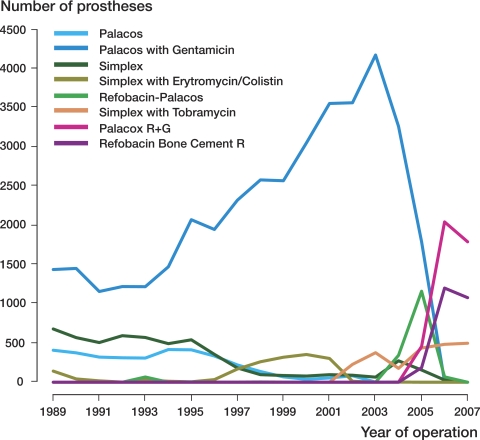
Time trends in the use of cement brands in Norway 1989–2007.

During the period 1987–2007, 4 of the 10 prosthesis brand combinations (cup/stem) constituted 85% of the operations (Table 1). Only 3 brands—Charnley, Exeter, and Titan—were used consistently throughout the period (Figure 2). Except for the monoblock Charnley prosthesis with a 22.225-mm head (47%), most stems had 28-mm modular heads (36%). All cups were UHMPWE (Table 1). Most of the caput prostheses were of stainless steel (63%), CoCr (26%), or alumina (10%). Overall, 28% of the operations involved males, the median patient age at operation was 73 years with 7.5% younger than 60 years, and 75% of patients were operated due to primary osteoarthritis. The cement Palacos with gentamicin was used in 64% of the operations (Table 2). Time trends were observed in the use of cement brands, where all 3 cement brands used in 2007 had been recently introduced (Figure 3). 2 of the prostheses, Kronos/Titan and Elite/Titan, had been used in few hospitals with at least 90% of the operations performed at the same hospital.

**Table 1.** Prosthesis characteristics for cemented prosthesis combinations in 62,305 total hip replacements, Norway 1987–2007

Prosthesis (cup/stem)	n	Manufacturer	Cup material **^d^**	Stem		Caput						
				Material **^f^** %				Diameter (%) **^h^**	Modular %	Modular brand (%) **^i^**
material	surface **^e^**	Steel	CoCr	Alum.	Other **^g^**
Charnley	29,577	DePuy	U	Steel	MV	100	0	0	0	22 (100)	0	–
Exeter	10,003	Stryker, Osteonics, Howmedica	U	Steel	P	59	0.2	40	0.0	28 (51)	97	Exeter (100)
Reflection **^a^**/	7,285	Smith & Nephew	U	CoCr	MR	0	89	6.3	4.4	28 (93)	100	Universal (95)
Spectron-EF												
Titan	6,250	Landos, DePuy	U	Ti	S	51	46	2.5	0	28 (70)	100	Landos (57)
Spectron/ITH **^b^**	2,355	Smith & Nephew	U	Ti	MS	0	97	3.1	0.1	32 (100)	100	Universal (96)
IP/SP (I, II) **^c^**	2,014	Waldemar Link	U	CoCr	M	0	99	1.1	0	28 (94)	90	SP II (100)
Contemporary/Exeter	1,571	Stryker, Howmedica	U	Steel	P	0.3	0.2	100	0	28 (100)	100	Exeter (99)
Kronos/Titan	1,303	Landos, DePuy	U	Ti	S	5.7	86	8.3	0	28 (100)	100	Fjord (85)
Elite/Titan	1,032	DePuy/Landos, DePuy	U	Ti	S	1.1	98	0.9	0	28 (98)	100	Fjord (97)
Reflection**^a^**/ITH**^b^**	915	Smith & Nephew	U	Ti	MS	0	95	0	5.3	28 (92)	100	Universal (92)

**^a^** Full brand name: Reflection Cemented All-Poly.

**^b^** The ITH femoral prosthesis is not in current use.

**^c^** Full brand name: Link IP/Lubinus SP I (n = 205) and Link IP/Lubinus SP II (n = 1,809).

**^d^** U – ultrahigh molecular weight polyethylene. 8 cups with unknown material.

**^e^** Surface: M – matt; MV – matt vaquasheen; MR – matt, roughened proximally; P – polished; S – smooth; MS – matt, sandblasted

**^f^** Unknown material in 981 modular heads.

**^g^** Zirconium (n = 334) and Oxinium (n = 37).

**^h^** The most commonly used caput diameter (mm). Information was not available in 184 cases.

**^i^** The most common caput prosthesis brand among modular stems. Information was not available in 61 cases.

**Table 2.** Patient and procedure characteristics for cemented prosthesis combinations in 62,305 total hip replacements, Norway 1987–2007

		No. of hospitals with						Cement brand (%)				
Prosthesis (cup/stem)	n	n > 10	n > 250	Men %	< 60 years %	Median age	OA % **^c^**	Palacos	Palacos G **^d^**	Simplex	Simplex E/C or T **^d^**	Palacos types **^e^**
Charnley	29,577	49	35	28	8.2	73	73	9.8	78	3.1	0.1	8.8
Exeter	10,003	17	10	29	8.6	72	79	0.7	26	33	33	7.7
Reflection **^a^**/Spectron-EF	7,285	16	10	28	7.9	74	80	0.4	65	4.8	4.4	25
Titan	6,250	19	6	27	2.2	75	75	9.2	78	1.8	0.1	11
Spectron/ITH	2,355	4	2	30	6.9	72	75	7.0	38	52	2.8	0.2
IP/SP (I, II) **^b^**	2,014	12	2	30	6.3	74	76	4.9	47	6.2	0.0	42
Contemporary/Exeter	1,571	10	1	33	12	72	83	0	19	3.9	9.8	67
Kronos/Titan	1,303	4	1	25	9.4	75	66	0.1	78	0	0	22
Elite/Titan	1,032	3	1	30	0.8	75	82	0	70	0	0	31
Reflection^a^/ITH	915	6	1	30	5.0	74	75	0	92	6.7	0.7	0.9
Overall	62,305	74	58	28	7.5	73	75	6.2	64	9.8	6.3	14

**^a^** Full brand name: Reflection Cemented All-Poly.

**^b^** Full brand name: Link IP/Lubinus SP I (n = 205) and Link IP/Lubinus SP II (n = 1,809).

**^c^** OA: osteoarthritis of the hip; information was not available on primary diagnosis in 400 cases.

**^d^** G: gentamicin; E/C: erythromycin/colistin; T: tobramycin

**^e^** Palacos types: Refobacin-Palacos (n = 1,652), Palacos R+G (n = 4,278), and Refobacin Bone Cement R (n = 2,458).

### Cemented THAs 1987–1997 (with follow-up until 2007)

**Figure 4.**
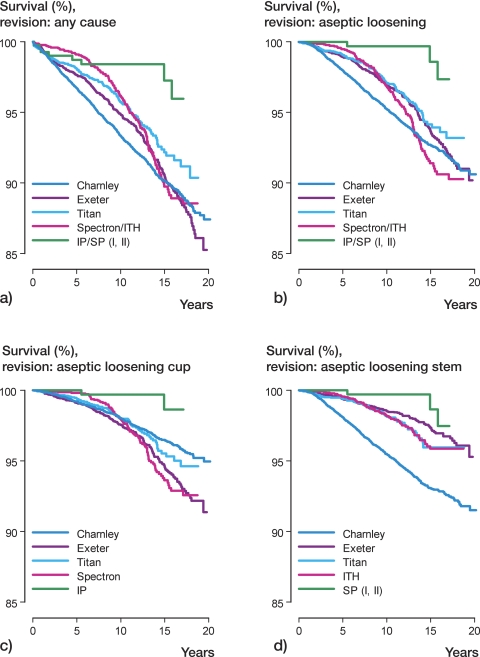
Prosthesis survival with revision of either cup or stem for any cause (a), revision of either cup or stem due to aseptic loosening (b), revision of cup due to aseptic loosening (c), or revision of stem due to aseptic loosening, (d) as endpoint, for 5 cemented prostheses operated 1987–1997 with follow-up through 2007.

5 of the 10 prosthesis cup/stem combinations had been used in more than 250 operations during the years 1987–1997 (Table 3), totaling 24,728 THAs. After 18 years, 11.3% (95% CI: 10.6–12.1) of these were revised for any cause, 8.4% (7.7–9.1) due to aseptic loosening, 5.3% (4.7–5.9) due to aseptic loosening of the cup, and 5.9% (5.3–6.4) due to aseptic loosening of the stem. We observed that all prosthesis combinations inserted during the period 1987–1997 had similar or better early survival (all causes of revision) than the Charnley (Table 4). However, beyond 10 years of follow-up, revision rates were higher for Exeter (RR = 1.4; 95% CI: 1.1–1.8) and Spectron/ITH (RR = 1.7; 1.3–2.2) (Table 4), indicated by a steeper decline of the survival curve beyond 10 years for these prostheses compared to that of the Charnley (4a). There were similar findings with revision due to aseptic loosening as endpoint, although beyond 10 years only Spectron/ITH (RR = 1.9; 1.4–2.5) had a statistically significantly higher revision rate compared to the Charnley (Figure 4b, Table Figure 4). With revision due to aseptic loosening of the cup, we observed no statistically significant differences between the 5 cups with follow-up of up to 10 years, but beyond 10 years revision rates were higher for the Exeter (RR = 1.8; 1.3–2.6) and the Spectron (RR = 2.4; 1.7–3.4) cups compared to the Charnley (4c, Table Figure 4). The same results were obtained when 2,059 metal-backed Exeter cups were excluded from the analysis. Although Titan, ITH, and SP stems had lower revision rates due to aseptic loosening than Charnley with short follow-up, we observed no statistically significant differences to the Charnley beyond 10 years. In contrast to the results for cups, the Exeter stem performed better than the Charnley throughout follow-up (RR = 0.4; 0.3–0.5) (4d, Table Figure 4).

**Table 3.** Median follow-up ^a^ and number at risk, for total hip arthroplasties inserted 1987–1997 (follow-up until 2007) and 1998–2007

		1987–1997				1998–2007	
		Number at risk by years after operation				Number at risk by years after operation	
Prosthesis (cup/stem)	Median FU (years)	0	10	18	Median FU (years)	0	8
Charnley	11	14,842	8,633	805	5.1	14,735	2,280
Exeter	12	3,934	2,562	326	4.1	6,069	653
Reflection **^b^**/Spectron-EF	10	66	48	0	3.6	7,219	242
Titan	11	3,205	1,758	92	4.5	3,045	461
Spectron/ITH	12	2,019	1,239	96	7.6	336	130
IP/SP (I, II) **^c^**	11	413	240	27	2.2	1,601	160
Contemporary/Exeter	0	0	0	0	1.3	1,571	0
Kronos/Titan	0	0	0	0	4.4	1,303	187
Elite/Titan	10	11	4	0	3.8	1,021	0
Reflection**^b^**/ITH	10	238	138	0	6.4	677	185
Overall	11	24,728	14,622	1,346	4.2	37,577	4,298

**^a^** Median follow-up (FU) calculated using the reversed Kaplan-Meier method.

**^b^** Full brand name: Reflection Cemented All-Poly.

**^c^** Full brand name: Link IP/Lubinus SP I (n = 205) and Link IP/Lubinus SP II (n = 1,809).

**Table 4.** Cox regression results ^a^ for cemented prosthesis brand combinations inserted 1987–1997 with follow-up until 2007 (n = 24,728)

	0–20-year follow-up				Revision %			6–10-year follow-up			11–20-year follow-up		
Cause of revision Prosthesis	No. revised	RR	95% CI	p-value	8-year	18-year	95% CI	No. revised	RR	p-value	No. revised	RR	p-value
All causes													
Charnley	1,141	1			5.3	12	11–13	406	1		242	1	
Exeter	330	0.9	0.8–1.1	0.3	3.9	12	10–15	102	0.8	0.1	124	1.4	0.02
Titan	146	0.7	0.6–0.8	< 0.001	3.0	9.7	7.2–12	51	0.7	0.01	39	1.0	1.0
Spectron/ITH	135	0.8	0.7–1.0	0.02	2.2	11	9.3–14	49	0.8	0.2	67	1.7	< 0.001
IP/SP (I, II) **^b^**	8	0.3	0.1–0.6	< 0.001	1.6	c	c	1	0.1	0.02	2	0.3	0.1
Aseptic loosening													
Charnley	851	1			3.6	8.6	7.8–9.5	340	1		203	1	
Exeter	219	0.8	0.6–1.0	0.03	2.1	8.8	6.9–11	74	0.7	0.01	96	1.3	0.1
Titan	100	0.7	0.5–0.8	< 0.001	1.8	6.8	5.1–8.9	43	0.7	0.02	32	1.0	1.0
Spectron/ITH	119	0.9	0.7–1.1	0.4	1.7	9.7	7.8–12	46	0.9	0.5	62	1.9	< 0.001
IP/SP (I, II) **^b^**	3	0.1	0.0–0.6	0.001	0.3	c	c	1	0.1	0.03	2	0.4	0.2
Aseptic loosening of cup													
Charnley	417	1			1.4	4.5	3.9–5.2	152	1		136	1	
Exeter	188	1.4	1.1–1.9	0.01	1.7	7.6	5.7–9.7	59	1.3	0.2	88	1.8	< 0.001
Titan	76	1.1	0.8–1.4	0.5	1.3	5.4	3.8–7.0	33	1.2	0.4	27	1.3	0.2
Spectron	90	1.4	1.1–1.8	0.003	0.9	7.5	5.7–9.2	33	1.5	0.05	53	2.4	< 0.001
IP **^b^**	2	0.2	0.1–0.8	0.03	0.3	c	c	1	0.3	0.2	1	0.3	0.3
Aseptic loosening of stem													
Charnley	789	1			3.5	7.8	7.0–8.6	321	1	1	178	1	
Exeter	106	0.4	0.3–0.5	< 0.001	1.1	3.9	2.7–5.1	35	0.3	< 0.001	40	0.5	0.002
Titan	63	0.5	0.4–0.6	< 0.001	1.2	4.0	2.9–5.2	26	0.5	< 0.001	21	0.7	0.2
ITH	59	0.5	0.4–0.6	< 0.001	1.3	4.1	3.0–5.3	25	0.5	< 0.001	24	0.8	0.3
SP (I, II)**^b^**	3	0.1	0.0–0.5	0.001	0.3	c	c	1	0.1	0.03	2	0.4	0.2

**^a^** Revision rate ratios (RRs) and revision percentages with adjustment for sex, age, diagnosis, and cement brand.

**^b^** Full brand name: Link IP/Lubinus SP I (n = 205) and Link IP/Lubinus SP II (n = 1,809).

**^c^** Less than 50 prostheses at risk.

### Cemented THAs 1998–2007

**Figure 5.**
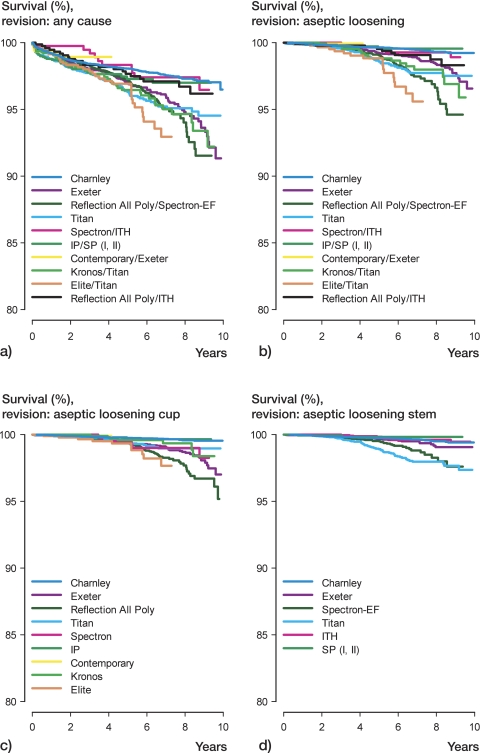
Prosthesis survival with revision of either cup or stem for any cause (a), revision of either cup or stem due to aseptic loosening (b), revision of cup due to aseptic loosening (c), or revision of stem due to aseptic loosening, (d) as endpoint, for 10 cemented prostheses operated 1998–2007.

**Figure 6.**
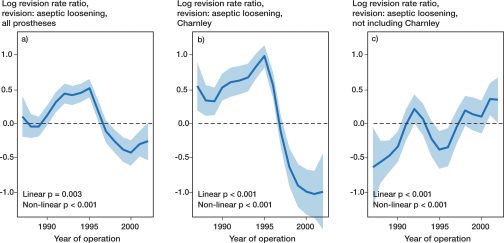
A graphical representation of the relationship between year of operation (1987–2002) and the log revision rate ratio with aseptic loosening as endpoint for all prostheses (a), Charnley prostheses (b), and all prostheses excluding Charnley (c). The graphs show 95% confidence intervals, together with results of a linear trend test and a test of non-linearity in the effect of year of operation. The graph is calibrated with the log revision rate ratio set to zero at the mean year of operation. The horizontal line shows the reference level.

All 10 prosthesis brand combinations had been used in more than 250 operations during this period (Table 3), totaling 37,577 THAs. The relative differences in prosthesis survival as compared to Charnley during this period (1998–2007) increased relative to our findings for the first time period (1987–1997). This was mainly due to a marked improvement in results for the Charnley from an estimated 8-year revision percentage of 5.3% (4.9–5.7) in the first period to 2.7% (2.3–3.1) in the last period with any revision as endpoint, and from 3.6% (3.3–4.0) to 0.7% (0.5–0.9) with revision due to aseptic loosening as endpoint. Except for Spectron/ITH and IP/SP, all prostheses had statistically significantly higher revision rates due to aseptic loosening than the Charnley (5b, Table Figure 5). 9 cups were investigated. Compared with the Charnley and with revision due to aseptic loosening of the cup as endpoint, beyond 5 years we found higher revision rates for all cups except for the IP (5c, Table Figure 6). Compared to the Charnley, the highest revision rates due to aseptic loosening were found for Reflection All-Poly (RR = 14; 7.2–28) and Elite (RR = 23; 7.8–67) cups. 6 stems were investigated and 2 of these, the Spectron-EF (RR = 6.1; 3.1 - 12) and Titan (RR = 5.5; 2.9–11) had inferior results to Charnley (5d, Table Figure 6).

**Table 5.** Cox regression results ^a^ with revision of either cup or stem as endpoint, for cemented prosthesis brand combinations inserted 1998–2007 (n = 37,577)

	0–10-year follow-up				Revision %		6–10-year follow-up		
Cause of revision Prosthesis	No. revised	RR	95% CI	p-value	8-year	95% CI	No. revised	RR	p-value
All causes									
Charnley	324	1			2.7	2.3–3.1	45	1	
Exeter	170	1.6	1.3–2.1	< 0.001	5.2	4.0–6.4	40	3.2	< 0.001
Reflection **^b^**/Spectron-EF	190	1.6	1.3–1.9	< 0.001	6.0	4.5–7.4	38	5.2	< 0.001
Titan	104	1.8	1.4–2.2	< 0.001	4.9	3.9–6.0	15	2.0	0.03
Spectron/ITH	11	1.1	0.5–2.3	0.8	2.6	0.6–4.6	5	2.0	0.2
IP/SP (I, II) **^c^**	38	1.4	1.0–2.0	0.08	3.0	1.9–4.1	3	0.3	0.3
Contemporary/Exeter	16	1.1	0.6–1.8	0.8	d	d	0	**^d^**	**^d^**
Kronos/Titan	48	1.9	1.4–2.6	< 0.001	5.4	3.6–7.2	11	3.4	< 0.001
Elite/Titan	35	2.1	1.5–3.0	< 0.001	d	d	11	9.8	< 0.001
Reflection**^b^**/ITH	23	1.2	0.8–1.9	0.4	3.3	1.8–4.8	6	1.8	0.2
Aseptic loosening									
Charnley	77	1			0.7	0.5–0.9	21	1	
Exeter	70	2.4	1.6–3.8	< 0.001	1.6	0.9–2.3	28	3.5	< 0.001
Reflection **^b^**/Spectron-EF	87	3.8	2.7–5.2	< 0.001	3.4	2.1–4.7	35	10	< 0.001
Titan	41	3.3	2.3–4.9	< 0.001	2.3	1.5–3.1	11	3.3	0.002
Spectron/ITH	8	1.4	0.6–3.7	0.5	0.7	0.0–1.5	5	2.1	0.2
IP/SP (I, II) **^c^**	5	0.8	0.3–2.1	0.6	0.4	-0.0–0.9	1	0.0	0.5
Contemporary/Exeter	1	0.7	0.1–4.8	0.7	d	d	0	d	d
Kronos/Titan	18	3.2	1.9–5.4	< 0.001	3.1	1.2–5.0	7	4.8	< 0.001
Elite/Titan	17	6.2	3.6–11	< 0.001	d	d	9	21	< 0.001
Reflection**^b^**/ITH	11	2.0	1.0–3.8	0.05	1.3	0.3–2.2	6	3.8	0.005

**^a^** Revision rate ratios (RRs) and revision percentages with adjustment for sex, age, diagnosis, and cement brand.

**^b^** Full brand name: Reflection Cemented All-Poly.

**^c^** Full brand name: Link IP/Lubinus SP I (n = 205) and Link IP/Lubinus SP II (n = 1809).

**^d^** Less than 50 prostheses at risk.

**Table 6.** Cox regression results ^a^ with revision of either cup or stem as endpoint, for cemented prosthesis brand combinations inserted 1998–2007 (n = 37,577)

		0–10-year follow-up				Revision %		6–10-year follow-up		
Cause of revision Prosthesis	No. primary	No. revised	RR	95% CI	p-value	8-year	95% CI	No. revised	RR	p-value
Aseptic loosening of cup										
Charnley	14,735	52	1			0.4	0.3–0.6	11	1	
Exeter	6,069	60	3.2	2.0–5.3	< 0.001	1.3	0.7–2.0	26	6.2	< 0.001
Reflection **^b^**	7,896	79	4.4	3.1–6.3	< 0.001	2.4	1.5–3.2	37	14	< 0.001
Titan	3,045	20	2.4	1.4–4.0	0.001	1.1	0.5–1.6	6	3.3	0.02
Spectron	336	6	3.3	1.0–11	0.05	1.0	-0.3–2.3	4	6.0	0.01
IP **^c^**	1,601	4	0.9	0.3–2.8	0.8	0.4	-0.1–0.8	1	0.0	0.6
Contemporary	1,571	1	1.3	0.2–9.5	0.8	d	d	0	d	d
Kronos	1,303	7	1.9	0.8–4.1	0.1	0.7	0.0–1.3	3	3.8	0.04
Elite	1,021	10	5.6	2.8–11	< 0.001	d	d	5	23	< 0.001
Aseptic loosening of stem										
Charnley	14,735	58	1			0.5	0.3–0.7	17	1	
Exeter	7,640	26	1.3	0.8–2.5	0.3	0.9	0.3–1.6	9	2.0	0.2
Spectron-EF	7,219	49	2.6	1.8–3.9	< 0.001	1.8	0.9–2.6	18	6.1	< 0.001
Titan	5,369	65	4.2	2.9–6.1	< 0.001	2.1	1.3–2.8	23	5.5	< 0.001
ITH	1,013	11	0.9	0.4–2.0	0.8	0.4	0.1–0.7	6	1.4	0.6
SP (I, II) **^c^**	1,601	2	0.5	0.1–2.1	0.4	0.2	-0.1–0.4	0	0.0	0.5

**^a^** Revision rate ratios (RRs) and revision percentages with adjustment for sex, age, diagnosis, and cement brand.

**^b^** Full brand name: Reflection Cemented All-Poly.

**^c^** Full brand name: Link IP/Lubinus SP I (n = 205) and Link IP/Lubinus SP II (n = 1809).

**^d^** Less than 50 prostheses at risk.

In this study, the Exeter was the most common prosthesis in 2007. With revision due to aseptic loosening of the cup as endpoint and the Exeter as reference (results not shown in tables), in the 6–10-year time interval we observed better results for Charnley (RR = 0.2; 0.1–0.4), and inferior results for Reflection All-Poly (RR = 2.3; 1.2–4.3) and Elite (RR = 3.6; 1.3–10) cups. With revision due to aseptic loosening of the stem as endpoint and the same time interval, we observed inferior results for Spectron-EF (RR = 3.1; 1.2–8.0) and Titan (RR = 2.8; 1.1–7.1) stems compared to the Exeter. The other brands of stem did not perform statistically significantly different from the Exeter.

Although it was used in over 1,500 operations and being the third most used prosthesis in 2007, the Contemporary/Exeter combination could not be evaluated due to the short follow-up time (median 1.3 years).

### Time trends

Extended Cox regression analyses showed a reduction in the risk of revision due to aseptic loosening since about 1995 (p for linearity = 0.003) (Figure 6). However, excluding the Charnley prosthesis, we observed an increase over time in revision risk due to aseptic loosening (p for linearity < 0.001) (Figure 6).

## Discussion

Overall survival for THAs inserted early (1987–1997) and late (1998–2007) showed short-term and long-term results that were satisfactory according to international standards (Aamodt et al. 2004). However, we observed clinically important differences between commonly used cemented prosthesis brands. The results for the Charnley prosthesis improved markedly over time, and in the last time period none of the other prostheses had better survival than the Charnley. The highest revision rates as compared with the Charnley were observed for the Reflection All-Poly/Spectron-EF and Elite/Titan combinations.

### Strengths and limitations

The study was based on information given to NAR by orthopedic surgeons since 1987. Ideally, the quality of prostheses should be evaluated in high-powered randomized clinical trials, but for practical and economic reasons (Black 1996) such trials are seldom performed for long-term comparison of hip implants. In register based studies, the findings may also be considered representative for a wider range of patients and surgeons. Registration completeness of hip replacements in the NAR is high (98%), both for primary operations and for revisions (Espehaug et al. 2006). Information on registration of selected variables has been validated based on data from one high-volume hospital, and few errors were observed in the registration of date of operation (1.1%) and in laterality (0.2%) (Arthursson et al. 2005). However, both the Danish and the Swedish hip arthroplasty registers have expressed caution regarding the validity of the registration of primary diagnoses (Pedersen et al. 2004, Kärrholm et al. 2007). Comparison of prosthesis brand survivorship in observational studies may give results confounded by patient and procedure characteristics. We observed distributional differences both in patient characteristics and in the use of cement brands. In our study, we limited the study population to patients with implants fixated with brands of cement that had reported satisfactory results (Espehaug et al. 2002). However, although shown to have comparable mechanical properties, handling curves and viscoelastic properties may not be identical for more recent cement preparations as compared to their predecessors (Dall et al. 2007). Furthermore, prosthesis survival for these cement preparations is largely unknown. We thus treated cement brand as a possible confounder in the statistical analyses along with sex, age, and diagnosis. Differences in survival may also be confounded by other factors not reported to the register, possibly surgeon-related or associated with time of the study. With this in mind, analyses were performed based on data from 2 time periods. One may also criticize the use of revision as endpoint, ignoring the high proportion of clinically and radiologically loose implants (Hulleberg et al. 2008). Although the total percentage of failure would be higher, however, it is unlikely that the relative differences between prosthesis brands would be affected.

### Prosthesis brands studied over 2 time periods: 1987–1997 and 1998–2007

Before 1998, the Charnley cup did well compared to others while the Charnley stem had inferior results—at least with short-term follow-up. Most Charnley cups were OGEE-flanged, which has been shown to give good cementation (Hodgkinson et al. 1993). The 22.225-mm head has also been shown to give lower wear rates than 28- and 32-mm heads, resulting in less aseptic loosening (Wroblewski et al. 2004). From about 1995, the results for both the Charnley cup and the Charnley stem improved markedly. The reason for this cannot be explained based on the data in the register, but it is known that from the mid-1990s onward, most surgeons using the prosthesis have been taught improved surgical technique. Another reason may be that the decrease in the use of Charnley prostheses might indicate that there may have been a selected group of dedicated surgeons that did not change to other brands of prosthesis.

Overall, the revision rates due to aseptic loosening were similar for Charnley and Exeter, but the long-term risk of aseptic loosening was higher for Exeter cups and lower for Exeter stems. This could be influenced by head size (51% for 28 mm and 39% for 30 mm, for the Exeter), sterilization procedures, the quality of polyethylene, design, surgical techniques, or a combination of these. This finding is corroborated by other studies showing excellent 12-year results for the Exeter stem, but not so for the Exeter cup (Williams et al. 2002, Lewthwaite et al. 2008). Inferior results have been reported with metal-backed Exeter cups in combination with the modular Exeter Universal stem (Hook et al. 2006). In our study, over 2,000 of the Exeter cups where metal-backed, but this had only marginal influence on the results.

Concern has been raised about the use of titanium stems in cemented prostheses (Jacobsson et al. 1995, Willert et al. 1996, Thomas et al. 2004). Our study showed that while the Titan stem did better than the Charnley in the first period, the opposite was true in the second period. This was not the case for the ITH stem, which is also made of titanium. While potentially important risk factors such as cementing technique and cement-mixing systems were not reported to the Norwegian Arthroplasty Register, the use of so-called modern cementing techniques was common in Norway throughout the last study period. The NAR has been informed that some of the titanium stems were inserted according to ‘the French paradox’ (Langlais et al. 2003), a method that was commonly used during the early years at some of the hospitals using titanium stems. Although criticized (Huiskes 1980, Anthony et al. 1990), the method has also been shown to give results similar to those using prostheses with a complete and thicker (≥ 2-mm) cement mantle (Skinner et al. 2003). Two French groups have also reported a stem revision rate of less than 1% due to aseptic loosening for cemented titanium stems designed to fill the medullary canal with the largest possible size (Nizard et al. 1992, LeMouel 1998).

The Spectron cup initially had better results than the Charnley cup, but this changed with longer follow-up—probably due to the 32-mm heads used with the Spectron cup (Wroblewski et al. 2004). Satisfactory short-term results have also been reported previously, based on data in the Norwegian Arthroplasty Register (Espehaug et al. 1995). Other studies have reported 4.1% revision after 11 years for the Spectron cup (Garellick et al. 2000).

We observed low revision rates for the IP/SP prosthesis (90% SP II stems). This is in accordance with those reported for the SP II prosthesis from other register-based studies with a 10-year revision rate due to aseptic loosening of 4.3% (Malchau et al. 2002). However, in our study only 240 IP/SP combinations had been followed for more than 10 years.

### Prosthesis brands studied for one time period: 1998–2007

The Reflection All-Poly cup and the cobalt-chrome Spectron-EF stem was the most common prosthesis combination in 2007 (with no restrictions regarding cement brand and including cups with highly crosslinked polyethylene). Compared with either the Charnley or the Exeter prosthesis, both the cup and the stem had higher revision rates. The 2007 annual report from the Swedish Hip Arthroplasty Register showed a revision rate (for any cause) at 10 years for this prosthesis combination of 8% (Kärrholm et al. 2008), which is similar to our findings of 6% at 8 years. An RSA study showed that cups with EtO-sterilized polyethylene (including the Reflection All-Poly cup) had almost twice the proximal and 3D femoral head penetration rates after 2 years as those with gamma-sterilized polyethylene (Digas 2005). The modular Spectron-EF stem was introduced in 1988, and in 1989 the roughness of the proximal part of the stem was increased. 2 publications have reported massive femoral osteolysis and metallosis for this stem and suggest that the addition of a rough surface to the Spectron stem has been detrimental to the long-term success of the prosthesis (Gonzalez Della Valle et al. 2006, Grose et al. 2006). Several studies have reported acceptable results (Garellick et al. 2000, Issack et al. 2003) for the monoblock Spectron stem that preceded the Spectron-EF stem, with a 16-year revision rate for aseptic loosening of 6.1% (Issack et al. 2003). It has been argued that a small stem in combination with a high offset will increase the risk of revision (Kärrholm et al. 2006). In our study, the number of Spectron-EF stems with this particular combination was small (n = 202), and when these were excluded we obtained similar results. The prosthesis combination Reflection All-Poly/ITH also had a higher revision rate due to aseptic loosening than Charnley, but this rate was not significantly different from that of the Exeter. We have not found any published reports for this prosthesis combination.

We could not find any published reports for the Kronos/Titan combination either, or for the Kronos cup. Our results should be interpreted with caution, as the Kronos/Titan combination was used mainly in one hospital.

Inferior results were noted for the Elite cup in combination with the Titan stem, as compared to either Charnley or Exeter. The Elite cup has the same manufacturer as the Charnley, and is the same except that it can be used with larger heads (98% with 28-mm heads). The finding for the Elite cup is in accordance with the concern raised by Walton et al. (2005) after observing a high degree of radiological loosening both for the acetabular and femoral Elite Plus components at a mean of 6 years postoperatively (Walton et al. 2005). Based on register data, inferior 5-year results have also been shown for the Elite/Charnley combination (Espehaug et al. 1995). However, it should be noted that the Elite/Titan combination was used mainly in one hospital.

With a median follow-up time of 1.3 years, the Contemporary/Exeter combination had the shortest follow-up. Even though the combination was one of the most commonly used prostheses in 2007, it could not be evaluated, and as far as we know no results have been reported from other studies.

### Conclusion

We observed clinically important differences between cemented prosthesis brands and identified inferior results for previously largely undocumented prostheses, including the commonly used prosthesis combination Reflection All-Poly/Spectron-EF. The study has further demonstrated the importance of long-term follow-up, as several of the prostheses with low short-term revision rates did not perform as well with longer follow-up. However, although we observed variation in prosthesis-specific survival, the overall results were satisfactory according to international standards.
